# Genome‑wide integrated analysis demonstrates widespread functions of lncRNAs in mammary gland development and lactation in dairy goats

**DOI:** 10.1186/s12864-020-6656-3

**Published:** 2020-03-23

**Authors:** Zhibin Ji, Tianle Chao, Zhaohua Liu, Lei Hou, Jin Wang, Aili Wang, Jie Zhou, Rong Xuan, Guizhi Wang, Jianmin Wang

**Affiliations:** Shandong Provincial Key Laboratory of Animal Biotechnology and Disease Control and Prevention, Shandong Agricultural University, 61 Daizong Street, Taian City, Shandong Province 271018 People’s Republic of China

**Keywords:** RNA-Seq, lncRNA, mRNA, Mammary gland, Goat

## Abstract

**Background:**

The mammary gland is a unique organ for milk synthesis, secretion and storage, and it undergoes cyclical processes of development, differentiation, lactation and degeneration. At different developmental periods, the biological processes governing mammary gland physiology and internal environmental homeostasis depend on a complex network of genes and regulatory factors. Emerging evidence indicates that lncRNAs have arbitrarily critical functions in regulating gene expression in many organisms; however, the systematic characteristics, expression, and regulatory roles of lncRNAs in the mammary gland tissues of dairy goats have not been determined.

**Result:**

In the present study, we profiled long noncoding RNA (lncRNA) expression in the mammary gland tissues of Laoshan dairy goats (*Capra hircus*) from different lactation periods at the whole-genome level, to identify, characterize and explore the regulatory functions of lncRNAs. A total of 37,249 transcripts were obtained, of which 2381 lncRNAs and 37,249 mRNAs were identified, 22,488 transcripts, including 800 noncoding transcripts and 21,688 coding transcripts, differed significantly (*p* ≤ 0.01) among the different lactation stages. The results of lncRNA-RNA interaction analysis showed that six known lncRNAs belonging to four families were identified as the precursors of 67 known microRNAs; 1478 and 573 mRNAs were predicted as hypothetical cis-regulation elements and antisense mRNAs, respectively. GO annotation and KEGG analysis indicated that the coexpressed mRNAs were largely enriched in biological processes related to such activities as metabolism, immune activation, and stress,., and most genes were involved in pathways related to such phenomena as inflammation, cancer, signal transduction, and metabolism.

**Conclusions:**

Our results clearly indicated that lncRNAs involved in responses to stimuli, multiorganism processes, development, reproductive processes and growth, are closely related to mammary gland development and lactation.

## Background

Long noncoding RNAs are a class of mRNA-like transcripts that have been operationally defined as mostly 5′-capped, polyadenylated and spliced, exhibiting lengths greater than 200 nt (nucleotide), and lacking ORFs (open reading frames) [1]. According to genomic localization of these RNAs with respect to nearby mRNAs, lncRNAs are divided into five loose categories: (1) sense lncRNAs, overlapped with one or more exons from another transcript on the same strand; (2) antisense lncRNAs, overlapped with one or more exons from another transcript on the other strand; (3) bidirectional lncRNAs, which initiate a similar expression pattern to its nearing mRNA counterpart on the other strand; (4) intronic lncRNAs, overlapped with one or more introns in another transcript; (5) and intergenic lncRNAs (lincRNAs), located in the interval between two protein coding genes on the same strand [2, 3]. Nevertheless, recent functional studies in mammals have found that lncRNAs serve as critical regulators in various biological processes by regulating gene expression at the epigenetic, transcription, and postttranscription levels. LncRNAs are widely involved in various important physiological and pathological processes, such as, cell differentiation, morphogenesis, gown, development and death, immunity responses, oncogenesis and cancer [4–6].

Dairy goats, an important domestic dairy breed worldwide, are developed mainly after birth, and their proliferation, differentiation, and apoptosis occur in cycles during puberty, pregnancy, lactation and involution. The mammary gland, as a special organ of dairy goats, is increasingly becoming an important model to study the fundamental physiology of development and homeostasis. To date, many studies have been performed to study ncRNAs (noncoding RNAs) of dairy goat mammary and have revealed a landscape of transcriptomics, miRomics, and proteomics [7–9]. However, to the best of our knowledge, studies of lncRNAomics of dairy goats have been reported only in ovary, hair follicle, skeletal muscle, hypothalamus, and skin pigmentation tissue [10–16], and there are no studies of the lncRNAomics of dairy goat mammary gland tissues in different lactating periods.

In our previous studies, miRNAomics and transcriptomics of dairy goat mammary glands at different lactating stages were analyzed using RNA sequencing [17, 18]. In the current study, for enhanced identification and characterization of the lncRNA profiles and function at the genome level, dairy goat mammary gland tissues in early lactation ((10th day after parturition), peak lactation (90th day after parturition) and late lactation (210th day after parturition) were collected, and the distribution, expression abundance and differential expression patterns of lncRNAs among different lactating periods of mammary gland development were tested and identified. Based on the data and bioinformatics, the action of lncRNAs on their target genes, the characteristics of mRNA transcripts, GO (gene ontology) enrichment and KEGG pathways (Kyoto Encyclopedia of Genes and Genomes) were also analyzed. Finally, a global regulatory network involving lncRNAs, miRNAs and their target genes was successfully constructed. From these results, we obtained a comprehensive characteristic of the lncRNA expression profile, and these results may help to elucidate the function of lncRNAs as members of the ceRNA (competing endogenous RNA) network in milk biosynthesis, transportation, and secretion or mammary gland development. It is of great significance to deeply study the internal balance physiological environment and regulation mechanism of goat mammary glands development and lactation.

## Results

### Transcript sequencing and assembly

A total of 88,938,426, 89,070,622 and 89,169,620 high-quality clean reads were obtained in the E, P and L libraries, respectively (E: early lactation, 10th day after parturition; P: peak lactation, 90th day after parturition; L: late lactation, 210th day after parturition). To further estimate the quality and coverage of the obtained transcripts, sequence alignment was performed on the reference genome of *Capra hircus*. For the different libraries, 94.85, 94.63 and 94.12% of the reads were mapped on the genome, including 82,892,999 unique matching reads and 1,415,064 multiple matching reads in the E library, 82,626,139 unique matching reads and 1,602,814 multiple matching reads in the P library, as well as 82,250,928 unique matching reads and 1,617,016 multiple matching reads in the L library. After assembly, a total of 39,863 transcripts were detected, including 37,249 transcripts identified as protein-coding mRNAs and 2614 transcripts identified as lncRNAs (Table 1).

**Table 1:** Data classification in different libraries obtained by RNA sequencing

Classification	E library		P library		L library	
	Number	Ratio	Number	Ratio	Number	Ratio
Total reads	92,762,706	100%	92,763,290	100%	90,500,394	100%
Clean reads	88,938,426	95.88%	89,070,622	96.02%	89,169,620	98.53%
Aligned to rRNA reads	55,386	0.06%	63,642	0.07%	60,186	0.07%
Mapped to genome	94.85%		94.63%		94.12%	
mRNA transcripts	31,099		30,120		32,254	
lncRNA transcripts	1937		1833		1984	

### Identification and genomic features of lncRNAs in the dairy goat mammary gland

To systematically identify lncRNAs in the whole genome wide, putative lncRNAs were identified based on their evolution conservation among different species, homology with known proteins, protein domains, and ORFs. After discarding the reads with adapters, more than 10% N substrate, low quality and rRNAs (ribosomal RNA), of the 267 million clean reads, 2381 potential lncRNAs were predicted by CPC, CNCI and PFAM with their genomics position, including 1777 known lncRNAs and 604 predicted novel lncRNAs, of which 667 were intronic lncRNAs, 536 were intergenic lncRNAs, 326 were antisense lncRNAs, 171 were sense lncRNAs and 42 lncRNAs belonged to bidirectional lncRNAs.

**Fig. 1:**
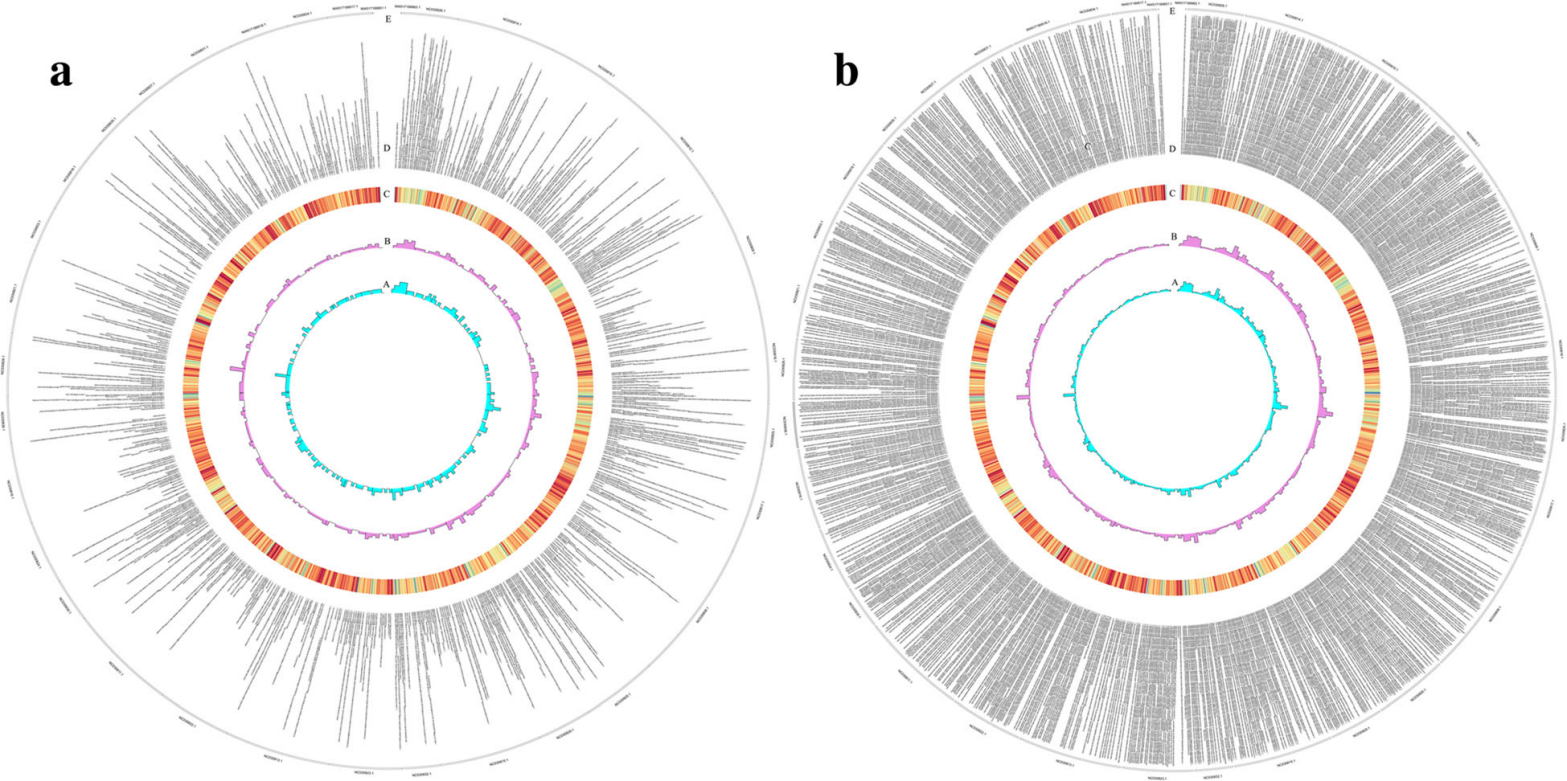
Chromosomal mapping of transcripts in the genome of *Capra hircus*. **a** Sequence distribution of lncRNAs. **b** Sequence distribution of mRNAs. From the inner to outer ring, A represents cDNA density. B represents sequences of lncRNA/mRNA density. C represents GC% (red represents high GC, green represents low GC). D represents location of transcripts on the genome. E represents chromosome

**Fig. 2:**
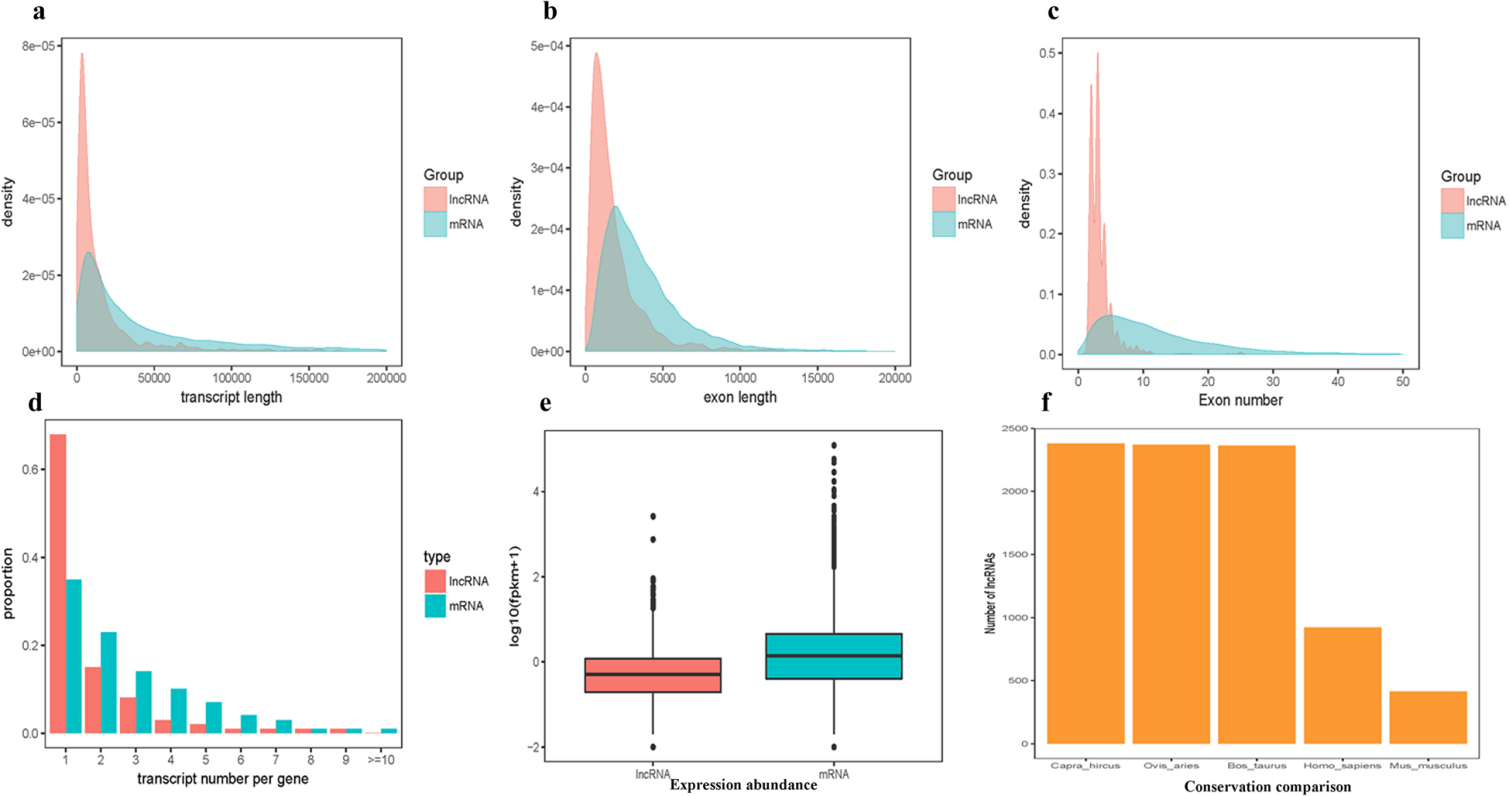
Comparison of the characteristic architecture between lncRNAs and mRNAs. **a** Transcript length distributions. **b** Exon length distributions. **c** Exon number distribution. **d** Transcript number Distributions. **e** Expression abundance distributions. **f** Conservation comparison of lncRNAs among different species

To explore the genomic features of these lncRNAs, the comparison of the characteristics was further performed with mRNAs. First, for the chromosome distribution, lncRNAs present a feature of arbitrary distribution on the 30 chromosomes, while the distribution of mRNAs had a certain preference (Fig. 1). Similar to other mammals, lncRNAs were found to have smaller lengths and fewer exons than protein-coding mRNAs. The lengths of the lncRNAs ranged from 417 to 602,577 nt, and most of them (86.3%) were approximately 400~3000 nt in length. The mean length was 19,722 nt, which was lower than the values observed for mRNAs (mean length = 129,518 nt) (Fig. 2a). On average, there were 3.44 exons that were 1901 bp long in lncRNAs, whereas mRNAs were 3777 bp long and had 12.62 exons (Fig. 2b and c). For transcript isoforms, there were 1.72 isoforms per lncRNA locus and 2 isoforms per mRNA locus. LncRNA loci possessed fewer transcript isoforms and lower protein-coding potential than protein-coding mRNA (Fig. 2d), and the median expression abundance of lncRNAs was significantly lower than those of mRNAs (Fig. 2e). The conservation of lncRNAs was also investigated, which were compared with those from sheep, cows mice and humans using BLASTN version 2.2. (E-value ≤1e-5, sequence identity > 20%). Of the 2381 lncRNAs in Laoshan dairy goats, 2371 lncRNAs were identified in sheep with a sequence identity greater than 80%, and in cows, humans and mice, they were 2358, 664 and 360, respectively (Fig. 2f).

### Family analysis and classification as miRNA precursors of lncRNAs

Based on evolutionary conservation, 59 known lncRNAs and 33 novel lncRNAs were classified into 53 different families in the Rfam database by sequence alignment. For known lncRNAs, the family with the most members was tRNA (transfer RNA, family accession: RF00005), which had nine members, while for novel lncRNAs, the family miR-154 had the highest number of members with 20 (Table S1).

Some lncRNAs may perform their functions as precursor molecules, to determine whether the identified lncRNAs were from the precursors of miRNAs. These lncRNA sequences were compared with miRNA precursors of *Capra hircus* in miRBase (Release 21, identity ≥90%). In all, 21 lncRNAs, including six known lncRNAs and 15 novel lncRNAs, were identified as precursors from 461 known miRNAs (Table S1).

### Differentially expressed lncRNAs and mRNAs in different lactating stages

**Fig. 3:**
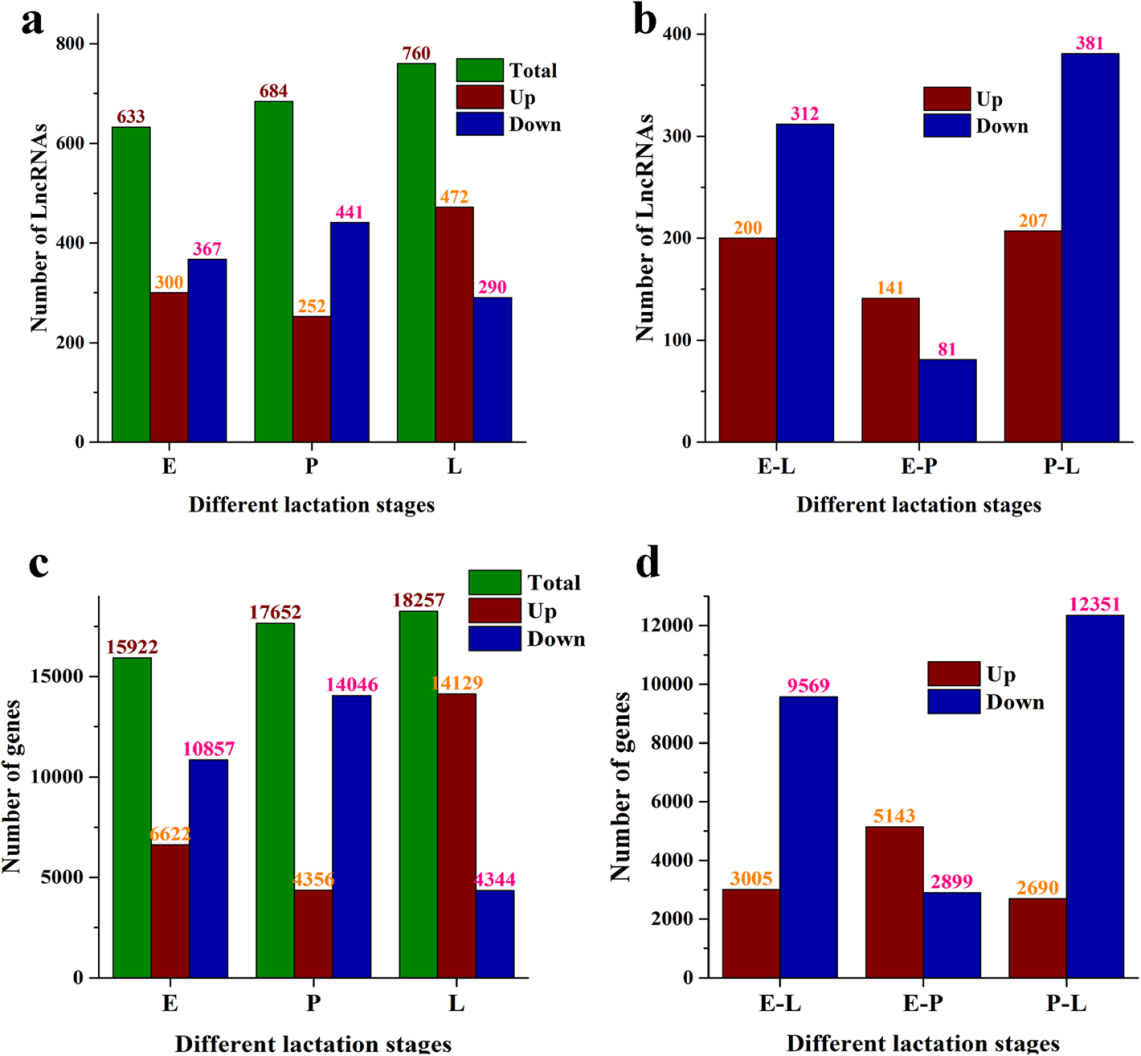
Distribution of lncRNAs and mRNAs in different lactation periods. **a** Total number of lncRNAs in different lactation periods. **b** The number of differentially expressed lncRNAs between different lactation periods. **c** Total number of mRNAs in different lactation periods. **d** The number of differentially expressed mRNAs between different lactation periods. E represents early lactation, P represents peak lactation, and L represents late lactation

In total, 2381 lncRNAs were identified and expressed in at least one of the three mammary gland lactating stages. In early lactation, compared with peak and late lactations, there were 633 differentially expressed lncRNAs, including 367 downregulated lncRNAs and 300 upregulated lncRNAs. Between early and peak lactation, 222 lncRNAs were differentially expressed, and between early and late lactation, 512 were differentially expressed. In peak lactation, compared with early and late lactations, 684 lncRNAs were differentially expressed. Compared with late lactation, 588 lncRNAs were differentially expressed, including 207 upregulated lncRNAs and 381 downregulated lncRNAs. In late lactation, a total of 760 lncRNAs were differentially expressed, including 290 downregulated and 472 upregulated lncRNAs, respectively (Fig. 3a and b, Table S2). Similar to lncRNAs, 18,697 mRNA transcripts were differentially expressed among different stages, and for early lactation, compared with peak and late lactations, a total of 15,922 transcripts were differentially expressed, including 6622 upregulated transcripts and 10,857 downregulated transcripts. Between early and peak lactations, 8042 transcripts were differentially expressed, and between early and late lactations, 12,574 were differentially expressed. In peak lactation, the number of differentially expressed transcripts was 17,652, with 4356 transcripts upregulated and 14,046 transcripts downregulated, respectively. Between peak and late lactations, 15,041 differentially expressed transcripts were detected. As for late lactation, 18,257 transcripts were differentially expressed, with 14,129 transcripts upregulated and 4344 transcripts downregulated (Fig. 3c and d, Table S3).

To further understand the functions of lncRNAs at the transcriptional or posttranscriptional levels, the antisense, up/downstream, and cis-regulated mRNAs were analyzed. A total of 883 lncRNAs were found transcribed near their mRNAs neighboring less than 10 kb, and 660 lncRNAs were combined with antisense mRNAs, including 2046 mRNAs. Of these RNAs, 573 lncRNAs and 1237 mRNAs were differentially expressed in mammary gland development among different lactating stages (Table S4 and S5).

### Function prediction of lncRNAs and corresponding genes

**Fig. 4:**
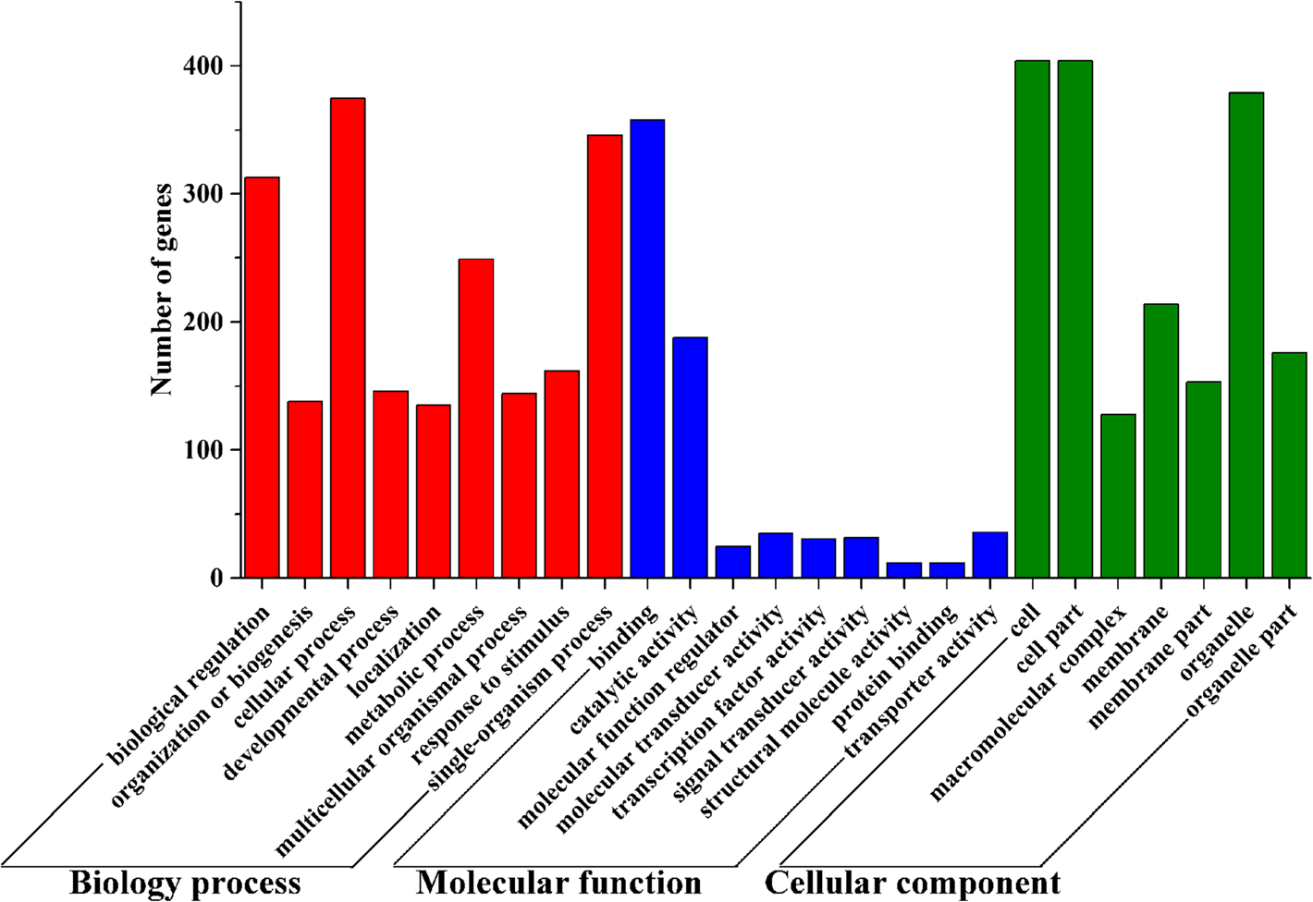
GO annotation enrichment of related genes of lncRNAs

In the present study, we used BLAST and RNAplex to predict the functions of lncRNAs and the corresponding genes. A total of 1478 genes were detected, of which 489 genes were annotated in biological processes; specifically, 375 genes were involved in cell processes, followed by single-organism processes, biological regulation, metabolic processes, response to stimulus, developmental processes, multicellular organismal processes, organization or biogenesis, and localization, and the number of genes in these classifications was 346, 313, 249, 162, 146, 144, 138, 135, respectively. For molecular function, there were 450 nonredundant genes, and the most three enriched classifications were binding activity, catalytic activity and transporter activity, and their corresponding gene numbers were 358, 188 and 36, respectively. For the cellular component, a total of 490 genes were classified into, and most genes were clustered into cell, cell part, organelle, membrane, organelle part, membrane part, and macromolecular complex; their corresponding numbers of genes were 404, 404, 379, 214, 176, 153, and 128, respectively (Fig. 4, Table S6).

**Fig. 5:**
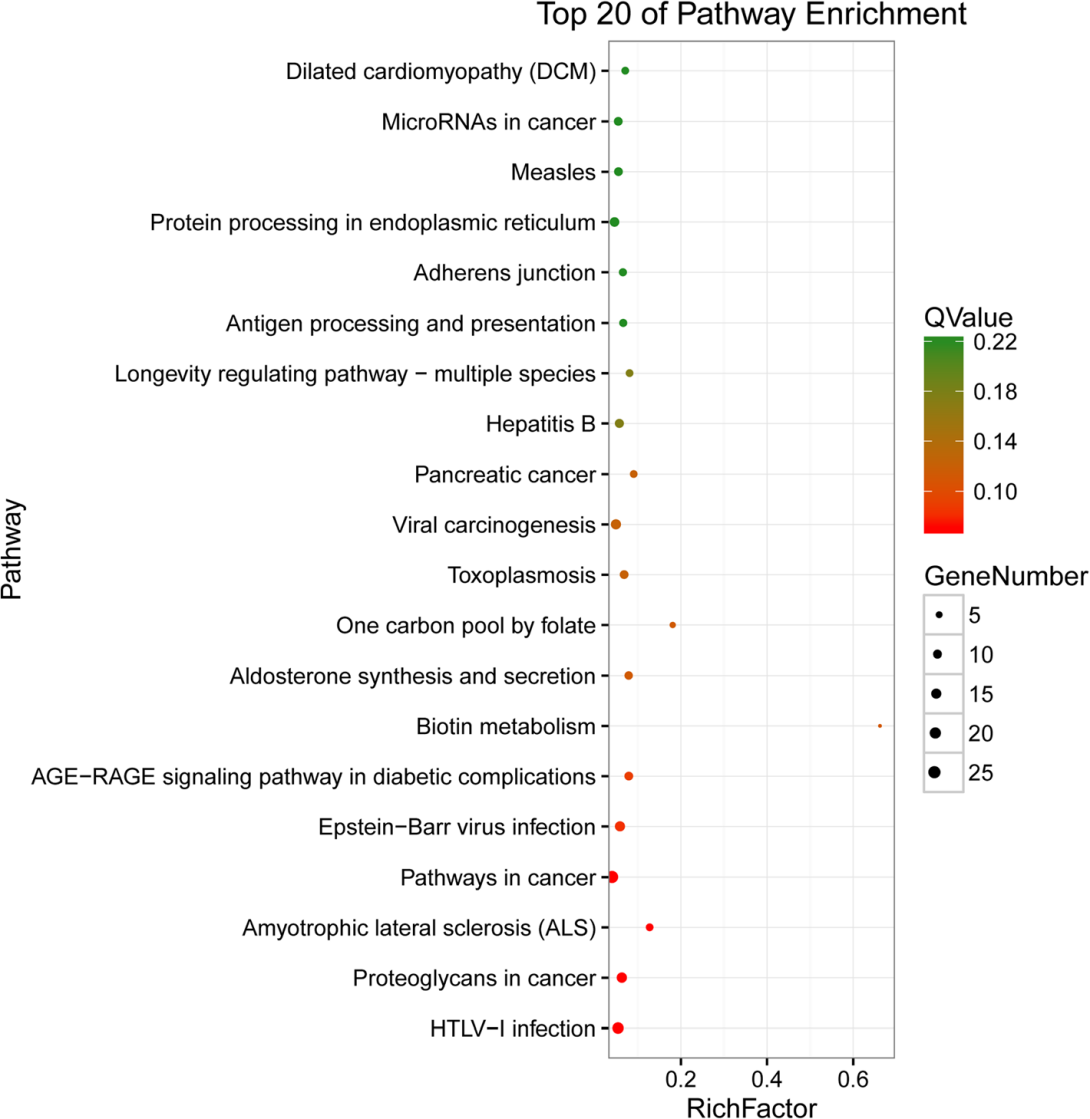
KEGG pathway enrichment analysis of related genes for lncRNAs. Different colors represent different *q*-values, and the size of the dots indicates the number of enriched genes

To further understand the biological functions of the identified lncRNAs and their genes, KEGG pathway analysis was also carried out (Fig. 5, Table S7). The results indicated that 33 pathways were significantly enriched with more than 10 genes. Many pathways are related to inflammation, cancer, signal transduction and metabolism. The top five most enriched pathway terms with the highest *P*-values were HTLV-I infection, proteoglycans in cancer, amyotrophic lateral sclerosis (ALS), pathways in cancer, and Epstein-Barr virus infection. The top five pathway terms with the most genes were pathways involved in cancer, HTLV-I infection, endocytosis, MAPK and PI3K-Akt. The 10 genes involved in these pathways were PIK3CB, PRKACA, TNF, ADCY5, MAPK8, EGFR, LOC108635162, ATF4, BCL2, and CACNA1S, which were all involved in more than 20 pathways.

### Construction of regulation networks

**Fig. 6:**
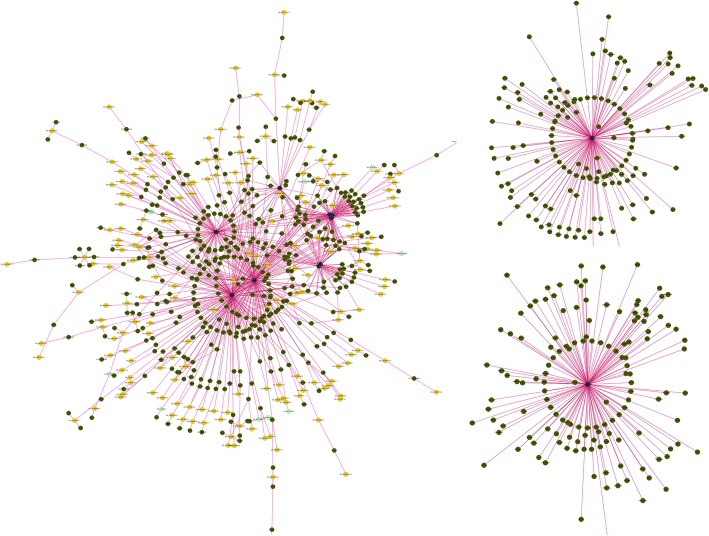
LncRNA-miRNA-mRNA coexpression network. Circles with yellow colors represent lncRNAs, circles with green colors represent genes, and circles with blue colors represent miRNAs. Purple lines indicate the regulation of different pairs

To further explore the important role of lncRNA in ceRNA networks, the potential premiRNAs in miRBase were detected using BLAST software; 11 miRNAs were obtained, which corresponded to six lncRNAs as well as their premiRNAs and mature miRNAs. MiRNAs were further verified by miRPara software, and 438 nonredundant target genes were predicted by TargetScan online software. A total of 537 lncRNAs with a read number greater than 10 were screened, including up- or downstream target genes or antimRNAs, and 1238 mRNAs were identified from differentially expressed mRNAs, which were up- or downstream of lncRNAs or their antisense chains. Finally, based on the miRNAs, differentially expressed lncRNAs and related mRNAs, 1290 pairs of factors with potential regulatory relationships were selected and used for the ceRNA regulation network construction (Fig. 6, Table S8). The results of further core model scoring analysis indicated that, two core models, miR-1247 and miR-2284r, played important regulatory functions in the ceRNA network via connecting lncRNAs and mRNAs.

## Discussion

Increasingly numerous studies have opened the door to our understanding of noncoding small RNA molecules, however, lncRNA research is in its infancy, and the functions of most lncRNAs have not been determined. With the development of high-throughput sequencing technology, an increasing number of lncRNAs have been identified, and their functions have also become increasingly detailed [19, 20].

In the present study, mammary gland tissues in early, peak and late lactation periods from five dairy goats were selected, and sequenced based on the Illumina HiSeq™ 2000 platform. To better connect with our previous study [17, 18], and the integrated analysis of multiomics data in the next step, the method of mixing pools of samples in the same period was also adopted in the current study. According to the quality control results, the construction and sequencing were successful, high quality data were obtained in three libraries, and 2381 lncRNAs and 37,249 mRNAs were identified.

LncRNAs, as a class of long-chain noncoding RNAs, exhibit certain deviations in their expression abundance, chromosome distribution, sequence length, and number of exons compared to mRNAs (Fig. 1 and Fig. 2). The spatiotemporal expression profiles showed that lncRNAs may have extensive physiological and biochemical regulatory functions [21, 22]. Among the 2381 lncRNAs, 800 were differentially expressed among different lactation stages. The number of lncRNAs identified in different lactation stages was also different, and some specifically expressed lncRNAs were found at a particular stage (Fig. 3, Table S2), Fifty-nine lncRNAs were specifically expressed in early lactation, 113 lncRNAs were specifically expressed in late lactation, and 23 lncRNAs were specifically expressed in peak lactation. This finding indicates that lncRNAs may play a unique role in the development and lactation stages of dairy goat mammary gland tissues. These characteristics indicate the functional diversity of lncRNAs, which may perform different important physiological regulatory roles in mammary gland development and lactation. Other studies, such as those conducted on bovines, have attempted to identify and characterize the expression of lncRNAs in peak, late and dry lactation stages [23, 24], and lncRNAs, as an important posttranscriptional regulators, participate widely in the regulation pathways related to the development and lactation of mammary glands. Therefore, an extensive investigation of the functions of lncRNAs will provideus with a clear understanding of the lncRNA word in various biological processes.

Initially, lncRNAs were considered “transcriptional noise” without any biological function [25]. Along with the increasing research investigating lncRNAs, a growing body of evidence suggests that lncRNAs play important regulatory roles in various physiological activities [26, 27]. However, due to a lack of corresponding databases, current methods, such as the analysis of sequence characteristics, molecular free energy, or secondary structure, for predicting the specific functions of lncRNA genes are still imperfect, and the biological functions of the majority of lncRNAs remain vague. Fortunately, many studies have been performed to find their associated up/downstream mRNAs and antisense mRNAs related to lncRNAs. Functional annotation and enrichment are considered an effective way to reveal the biological significance and biological processes or functions of most enriched genes [28–30]. In this study, based on these basic principles, 2046 genes were scanned as apcGenes and antisense mRNAs for 883 lncRNAs, combined with the differentially expressed lncRNA, mRNAs and predicted pre-miRNA, as well as their interaction, and 1478 genes were detected and further served for GO enrichment and KEGG pathway analyses. From these results, it is clearly indicated that most lncRNAs together with their apcGenes and antigens participate widely in diverse biological regulation pathways, such as HTLV-I infection, and proteoglycans in cancer., These physiological processes are closely related to cell proliferation and apoptosis, and they are the same as those involved in the physiological cycle of mammary gland development, These genes may play a leading role in the processes regulating mammary gland development (Figs. 4 and 5, Tables S5, S6 and S7).

Many previous studies have clearly confirmed that miRNAs, as vital regulation factors, are widely involved in various biological processes by changing the expression of targeted mRNAs, while studies in recent years have also shown that lncRNAs, having the effect of sponge adsorption for miRNAs, can shield the inhibition or degradation of target genes and rapidly upregulate their expression by competing to bind the miRNAs, which shows a new mechanism of ceRNA gene repression regulation [31, 32]. In the current study, the following factors were screened to construct the ceRNA regulation networks: lncRNAomic and transcriptomic data, cis- and trans-mRNAs, up- and downstream mRNAs of lncRNAs, target mRNAs of miRNAs, and the bioinformatics conjoint differentially expressed lncRNAs and mRNAs. As a result, 537 lncRNAs, 11 miRNAs and 1238 mRNAs were selected to construct the ceRNA regulation networks (Fig. 6, Table S8), and two core models, miR-1247 and miR-2248r, contained the largest number of interaction regulatory elements, indicating that they may be important regulatory factors in the lactation physiology of dairy goat mammary glands. In further studies, the corresponding experiments will be performed to validate their interaction and functions.

In summary, based on these studies, we found that lncRNAs, as core regulatory elements, play important roles in mammary gland development and lactation physiology in dairy goats. These findings provided theoretical references for further research about their inheritance, improvement and gene regulation.

## Conclusions

In current study, based on the three libraries from early, peak and late lactations of dairy goat mammary gland tissues, 37,249 transcripts were obtained, including 2381 lncRNAs and 37,249 mRNAs, of which, 800 noncoding transcripts and 21,688 coding transcripts differed significantly (*p* ≤ 0.01) among different lactating periods. The results of bioinformatics analysis in ceRNA networks indicated, that six known lncRNAs were identified as precursors of 67 known miRNAs, and 1478 and 573 mRNAs were predicted as hypothetical cis-regulation elements and antisense mRNAs, respectively. Based on GO and KEGG analysis, 1290 pairs of factors were selected to construct the ceRNA networks. These findings support that lncRNA, as a critical regulatory factor, widely participates in mammary gland development and lactation in dairy goats. Our results may enlarge the foundation for the further investigation of the core molecular functions of lncRNA in the physiological regulation of domestic animals, but may also provide some theoretical reference for further studies about their inheritance, improvement and gene regulation”.

## Methods

### Animal sample preparation and total RNA extraction

Five healthy Laoshan dairy goats (4 years old, third lactation) from Qingdao Laoshan dairy goat primary farm (Shandong Province, China) were prepared, all goats had free food and water access, and they were housed with natural lighting prior to experiments. The mammary gland specimens in early (10th day after parturition), peak (90th day after parturition) and late (210th day after parturition) lactations, were collected surgically after general anesthesia by intramuscular injection of Xylazine Hydrochloride injection solution (Huamu Animal Health Products Co., Ltd. China). The mammary glands were sutured by surgery, sterilized regularly, and the goats were all released after natural recovery. All mammary gland specimens were only used for scientific study, and were not released.

Total RNA was individually extracted from the above fifteen samples using TRIzol reagent (Invitrogen, USA) according to the manufacturer’s references, their integrity and quality were tested by a 2100 Bioanalyzer (Agilent, USA) and the samples (RIN ≥ 7.7) were used for library construction.

### Library construction, deep sequencing and transcript assembly

Individual RNA samples collected from five dairy goat mammary gland tissues in the same lactating stage were thoroughly mixed, and used further for library construction using the Illumina TruSeq Stranded RNA Kit (Illumina, USA) according to the manufacturer’s recommendations, The samples were named the E library (early lactation), P library (high lactation) and L library (late lactation),. The sequencing works were performed on an Illumina HiSeq™ 2000 platform in Beijing Genomics Institute (BGI, Shenzhen, China).

To systematically profile and identify and quantitate transcripts of dairy goat mammary gland tissues in different lactating periods, we performed PE150 (paired-end 150) sequencing to obtain global transcripts at the whole-genome level. Approximately 50–70 million raw reads were scanned for every library after removing the 3′ adaptor sequences, the low-quality reads and the various impurities. Trimmomatic-0.36 [33] was used to discard the reads containing adapters, trimming the reads with a poly(N) substrates more than ≥10% and removing the low quality reads with a quality score less than 20, the remaining reads (named clean reads) were used for downstream analyses. All clean reads were mapped to the *Capra hircus* genome (v1.01, https://www.ncbi.nlm.nih.gov/genome/?term=GOAT) using TopHat v2.0.9, and then assembled using Cufflinks v2.1.1 [34] based on the reference genome of goat (https://www.ncbi.nlm.nih.gov/genome/?term=goat, GCF_001704415). These transcripts were also removed according to the following criterias: length less than 200 bp, only one exon, less than three reads. After the remaining transcripts of each sample were constructed, a set of transcripts was merged using Cuffmerge software to generate the corresponding transcriptome. Bowtie2 soft was used to scan the transcripts, and the rRNA, tRNA, snRNA (small nuclear RNA), snoRNA (small nucleolar RNA), premiRNA (precursor miRNA) and pseudogenes, were also recognized and removed in the next analysis. The expression abundance of all transcripts in each library was tested using Cuffdiff based on the FPKM (fragments per kilobase of transcript per million mapped reads). A *P value* < 0.05 was considered to indicate a significant difference among different samples.

### LncRNA identification, conservation analysis and coding potential prediction

A stringent filtering analysis pipeline was developed to identify potential lncRNAs from the assembled transcripts according to Wu et al., (2016) [35]: (1) transcripts that overlapped with any protein-coding exon in the sense orientation were removed; (2) transcripts with < 200 bp, a single-exon, read coverage < 0.8, and a FPKM < 0.1 were eliminated; (3) transcripts with predicted large ORFs (> 100 aa) were filtered out; (4) transcripts with predicted protein-coding potential were removed; (5) transcripts with similarity to known protein sequences in the Swiss-Prot database and known protein-coding domains in the Pfam (AB) database were discarded; (6) transcripts within the < 2 k scaffold-end range were excluded; and finally, (7) transcripts with aaaaa were retained as presumptive lncRNAs.

Phylogenetic analysis of lncRNAs and mRNAs was carried out by BLASTN (E-value ≤1e-5, sequence identity > 20%) with default parameters among *Capra hircus*, *Ovis aries*, *Bos taurus*, *Homo sapiens*, and *Mus musculus*. CPC (coding potential calculator) [36], CNCI (coding-noncoding index) [37] and PFAM [38] were used to predict transcripts with the ability of encoding proteins. All transcripts without coding potential were retained.

### Family analysis and precursor prediction of lncRNAs

Infernal software [39] was used to align to the Rfam database. Based on the conservative sequence, the two-level structure and covariance model, the lncRNAs were classified into different families. We compared lncRNAs to miRBase to find potential miRNA precursors, and sequences with a comparison coverage greater than 90% were selected as postulated precursors.

### Differential expression and apcGene (adjacent protein-coding genes) analysis of lncRNAs

For differential expression, each lncRNA was identified by EdgeR version 3.0.8 [40] in the three libraries using an *FDR* no more than 0.05 and an absolute fold change no less than two as the screening conditions.

According to the location of the lncRNAs and protein-coding genes on the *Capra hircus* genome (v1.01), the nearest protein-coding genes located on the 5′ upstream or 3′ downstream of a lncRNA were named apcGenes. The apcGenes adjacent to each lncRNA at up- or down- stream positions within 10 kb were identified using BLAST. The complementary combination between antisense lncRNA and mRNA was identified by RNAplex software [41].

### GO and KEGG pathway analysis

All genes that were differentially expressed between different libraries combined with apcGenes and postulate target genes were annotated and enriched in the GO database using GOseq package v1.18.0 for R [42]. Significant pathways were enriched by KOBAS v2.0 [43] in the KEGG pathway database. A Benjamini-corrected modified Fisher’s statistical method was used to calculate the *P* values, and a *P-value* ≤ 0.05 was used as the critical value of significance for the enrichment analysis of GO and KEGG.

### Network construction

Based on the above comprehensive analysis results of the GO and KEGG of all DEGs (differential expression genes), apcGenes, and the predicted target genes of the lncRNAs, we selected genes and lncRNAs related to mammary gland development and lactation to construct the regulation network. Cytoscape v.3.2.1 software [44] was used to visualize the coexpression networks.

## Supplementary information

**Additional file 1: Figure S1.** Ethics approval and consent.

**Additional file 2: Table S1.** Family classification of lncRNAs in the Rfam database.

**Additional file 3: Table S2.** Differentially expressed lncRNAs between different lactation periods.

**Additional file 4: Table S3.** Differentially expressed transcripts between different lactation periods.

**Additional file 5: Table S4.** Differentially expressed mRNAs between different lactation periods.

**Additional file 6: Table S5.** Predicted apcGenes of lncRNAs.

**Additional file 7: Table S6.** GO annotation of related genes of lncRNAs.

**Additional file 8: Table S7.** KEGG pathways of related genes of lncRNAs.

**Additional file 9: Table S8.** Regulatory relationship between lncRNAs, miRNAs and mRNAs.

## Data Availability

All the data supporting the findings can be available in manuscript and additional files. All the raw data has been deposited to the GEO (Gene Expression Omnibus) and SRA (Sequence Read Archive) in NCBI, with the accession number of GSE135930 (https://www.ncbi.nlm.nih.gov/geo/query/acc.cgi?acc=GSE135930) and SRP218662 https://www.ncbi.nlm.nih.gov/sra?term=SRP218662, respectively.

## Abbreviations

lncRNAs: Long noncoding RNA
nt: Nucleotide
ORF: Open reading frame
lincRNA: Intergenic lncRNA
ncRNA: Noncoding RNA
GO: Gene ontology
KEGG: Kyoto Encyclopedia of Genes and Genomes
ceRNA: Competing endogenous RNA
PE: Paired-end
E: Early lactation
P: Peak lactation
L: Late lactation
snRNA: Small nuclear RNA
snoRNA: Small nucleolar RNA
premiRNA: Precursor miRNA
FPKM: Fragments per kilobase of transcript per million mapped reads
apcGene: Adjacent protein-coding genes
DEGs: Differential expression genes
