# Supplementary figures and images for: Genome‑wide integrated analysis demonstrates widespread functions of lncRNAs in mammary gland development and lactation in dairy goats
Source: BMC Genomics. 2020 Mar 23;21:254. doi: 10.1186/s12864-020-6656-3 (PMC7092584; doi:10.1186/s12864-020-6656-3)

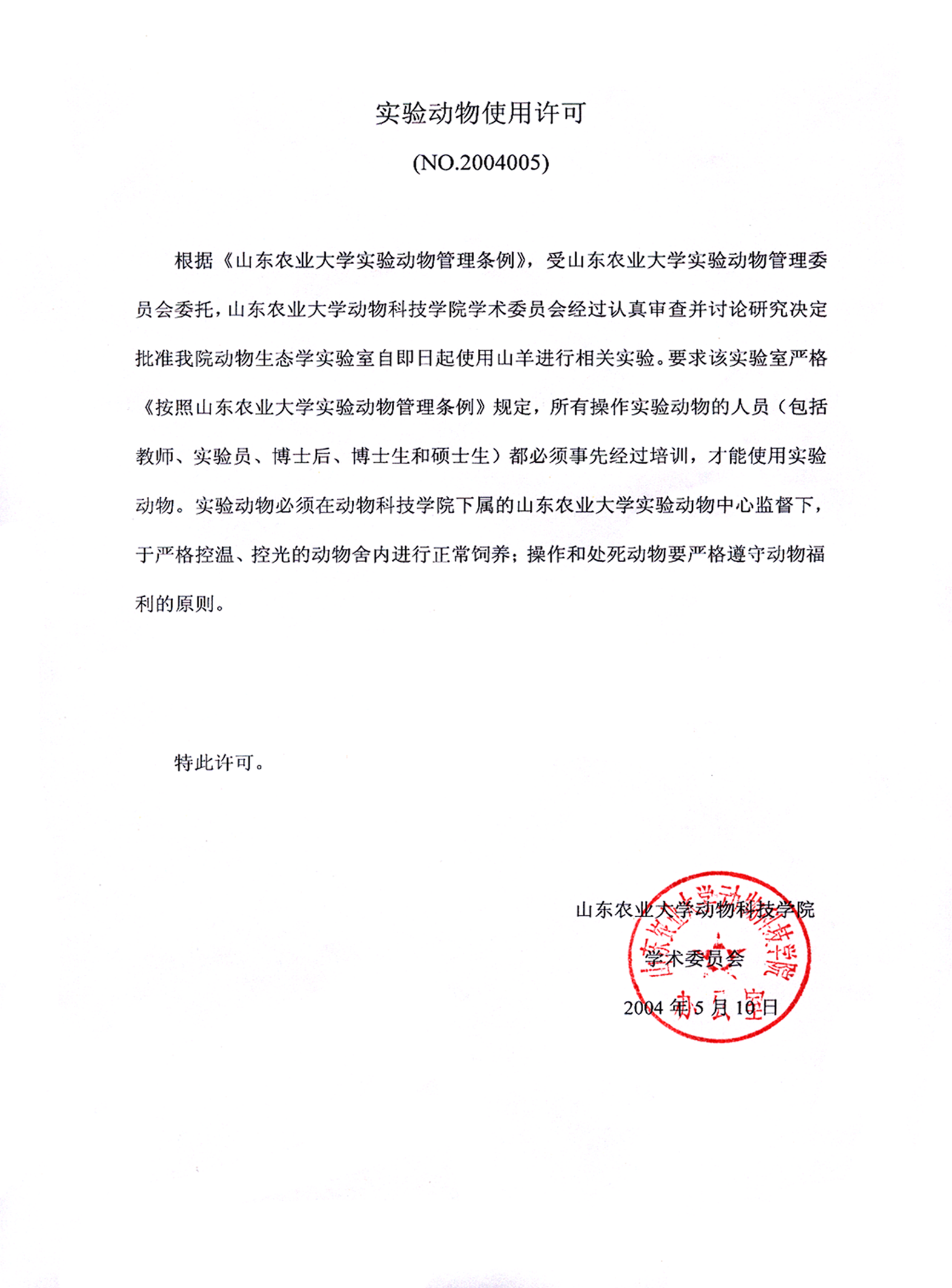

Supplement: Supplementary file 1 — Additional file 1: Figure S1. Ethics approval and consent. [file 12864_2020_6656_MOESM1_ESM.tif]
